# Pharmaceutical Pollution in Aquatic Environments: A Concise Review of Environmental Impacts and Bioremediation Systems

**DOI:** 10.3389/fmicb.2022.869332

**Published:** 2022-04-26

**Authors:** Maite Ortúzar, Maranda Esterhuizen, Darío Rafael Olicón-Hernández, Jesús González-López, Elisabet Aranda

**Affiliations:** ^1^Department of Microbiology and Genetics, Edificio Departamental, University of Salamanca, Salamanca, Spain; ^2^Ecosystems and Environment Research Programme, Faculty of Biological and Environmental Sciences, Finland and Helsinki Institute of Sustainability Science, University of Helsinki, Helsinki, Finland; ^3^Joint Laboratory of Applied Ecotoxicology, Korea Institute of Science and Technology Europe, Saarbrücken, Germany; ^4^University of Manitoba, Clayton H. Riddell Faculty of Environment, Earth, and Resources, Winnipeg, MB, Canada; ^5^Instituto Politécnico Nacional, Departamento de Microbiología, Escuela Nacional de Ciencias Biológicas, Mexico City, Mexico; ^6^Environmental Microbiology Group, Institute of Water Research, University of Granada, Granada, Spain; ^7^Department of Microbiology, Faculty of Pharmacy, University of Granada, Granada, Spain

**Keywords:** pharmaceutical active compounds, bioremediation, wastewater, mycoremediation, emerging contaminants, pharmaceutical pollution

## Abstract

The presence of emerging contaminants in the environment, such as pharmaceuticals, is a growing global concern. The excessive use of medication globally, together with the recalcitrance of pharmaceuticals in traditional wastewater treatment systems, has caused these compounds to present a severe environmental problem. In recent years, the increase in their availability, access and use of drugs has caused concentrations in water bodies to rise substantially. Considered as emerging contaminants, pharmaceuticals represent a challenge in the field of environmental remediation; therefore, alternative add-on systems for traditional wastewater treatment plants are continuously being developed to mitigate their impact and reduce their effects on the environment and human health. In this review, we describe the current status and impact of pharmaceutical compounds as emerging contaminants, focusing on their presence in water bodies, and analyzing the development of bioremediation systems, especially mycoremediation, for the removal of these pharmaceutical compounds with a special focus on fungal technologies.

## Introduction

In recent decades, the production and consumption of pharmaceutical products have rapidly increased with the development of medicine. Approximately 3,000 compounds are used as pharmaceuticals, and the annual production quantity exceeds hundreds of tons (Carvalho and Santos, 2016; Grenni et al., 2018). Anti-inflammatory drugs, antibiotics, and analgesics are the most common drugs used around the world. Consequently, the emergence of water-soluble and pharmacologically active organic micropollutants or pharmaceutical active compounds (PhACs) has gained much attention worldwide. Humans use a variety of these pharmaceuticals for their health in everyday life, but large quantities of these drugs are also used as veterinary medicine on farms around the world, to prevent and treat animal diseases and to increase economic benefits in intensive livestock (Blanco et al., 2017; Ekpeghere et al., 2017; Gros et al., 2019; Ramírez-Morales et al., 2021).

After ingestion, pharmaceuticals are excreted in urine and feces as active substances or metabolites (Sui et al., 2015; aus der Beek et al., 2016). These pharmaceuticals are present in both influent and effluent wastewater but can also be found in surface water bodies, including freshwater ecosystems and marine environments, as well as in groundwater due to effluent leachates generated under recharge conditions (Deo, 2014; Furlong et al., 2017; Ojemaye and Petrik, 2018; Reis-Santos et al., 2018; Fekadu et al., 2019; Letsinger et al., 2019; Zainab et al., 2020). The main concern is that conventional treatment plants are ineffective in removing some of these emerging contaminants (ECs), and new techniques are being sought and studied to achieve their total elimination, particularly advances in mycoremediation (Danner et al., 2019). The importance of the study of pharmaceuticals lies in the massive increase in their consumption worldwide, as well as in the environmental repercussions that this entails, including their recalcitrance in aquatic and terrestrial ecosystems. In the contexts of wastewater and bioremediation, pharmaceutical compounds are considered as ECs due to the lack of regulation for their environmental disposal, as well as the lack of information regarding their long-term effects on the environment (Dhangar and Kumar, 2020; Valdez-Carrillo et al., 2020; Chaturvedi et al., 2021b; Rathi et al., 2021), which remains unknown (Barber et al., 2015; Ahmed et al., 2017). The fact that some drugs are marketed without medical prescription or pre-registration and, therefore, are widely consumed worldwide, meaning that they are widely distributed in the environment (Gil et al., 2017), has contributed to this growing problem.

Considering pharmaceuticals as ECs and the continual production of new PhACs, this review aims to comprehensively present the pharmaceuticals commonly detected in water, surface and groundwater and their adverse environmental effects. Advances in bioremediation technologies, which can be used as add-on treatments in wastewater treatment plants (WWTPs) to reduce unprocessed pharmaceuticals released via effluent into the environment, are presented and critically discussed with an emphasis on mycoremediation.

## Common Pharmaceuticals Detected in Water (Surface and Groundwater)

**FIGURE 1:**
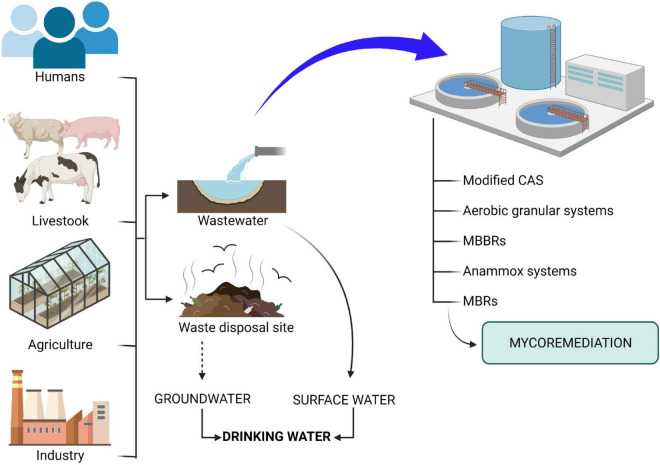
Pharmaceuticals route to a body of water and bioremediation technologies. (→): Direct contamination. (⇢): Contamination through different steps. The monitoring suggests that contamination accumulates in surface water and groundwater.

Pharmaceutical compounds that reach water bodies, both surface water and groundwater, came from a number of different sources (Figure 1). The first of these is urban wastewater, which contains a high load of pharmaceuticals from human excrement, and also the inadequate disposal of expired or unused drugs due to the scarce control in their management. Another major source of pharmaceuticals is agricultural and livestock waste, especially the latter, since in large farms for intensive livestock, animals are often fed with feed supplemented containing drugs and excreta are often used in agriculture as soil amendments, reaching groundwater by leaching (Kim et al., 2008; Barrios-Estrada et al., 2018). Effluents from the pharmaceutical industry are another important source, with high concentrations of pharmaceuticals being found due to discharges from factories in Asia, Europe and America, despite strict regulation of pharmaceutical production in Europe and the United States (Lin et al., 2008; Lin and Tsai, 2009; Phillips et al., 2010; Prasse et al., 2010; Sim et al., 2011; Cardoso et al., 2014). These industries are obliged to carry out treatment before discharge into the general urban sewer network (Lindberg et al., 2004; Brown et al., 2006).

Pharmaceuticals found in high concentrations in wastewater include non-steroidal anti-inflammatory drugs (NSAIDs), β-blockers ad psychoactive compounds, analgesics, antibiotics, endocrine disruptors, antiretroviral drugs, and drugs to treat cancer (Roberts and Thomas, 2006; Gros et al., 2010; Lian et al., 2017). These are the PhACs most commonly detected due to the analytical methods available and their resolution, although new methods for identifying these compounds are increasingly being developed (Pivetta et al., 2020; Zhang et al., 2020). Table 1 shows the worldwide distribution of the drugs most commonly found in water (Supplementary Figure 1).

**TABLE 1:** Types of pharmaceuticals and concentrations reported in countries worldwide.

Pharmaceutical type	Pharmaceutical	Max conc (ng/L)	Country	References
NSAIDs and analgesics	Naproxen	4,889	Mexico	Rivera-Jaimes et al., 2018
NSAIDs and analgesics	Acetaminophen	4,460	Mexico	Rivera-Jaimes et al., 2018
NSAIDs and analgesics	Diclofenac	1,398	Mexico	Rivera-Jaimes et al., 2018
NSAIDs and analgesics	Diclofenac	10,221	Saudi Arabia	Ali et al., 2017
NSAIDs and analgesics	Acetaminophen	2,346	Saudi Arabia	Ali et al., 2017
NSAIDs and analgesics	Ibuprofen	2,094.4	Brazil	Pereira et al., 2016
NSAIDs and analgesics	Acetaminophen	34.6	Brazil	Pereira et al., 2016
NSAIDs and analgesics	Diclofenac	19.4	Brazil	Pereira et al., 2016
NSAIDs and analgesics	Acetaminophen	48.74	Antartic Peninsula	González-Alonso et al., 2017
NSAIDs and analgesics	Diclofenac	15.09	Antartic Peninsula	González-Alonso et al., 2017
NSAIDs and analgesics	Ibuprofen	10.05	Antartic Peninsula	González-Alonso et al., 2017
NSAIDs and analgesics	Ibuprofen	414	South Korea	Kim et al., 2009
NSAIDs and analgesics	Ibuprofen	1,850	Vietnam	Tran et al., 2014
NSAIDs and analgesics	Diclofenac	1,630	Vietnam	Tran et al., 2014
NSAIDs and analgesics	Ketoprofen	1,620	Vietnam	Tran et al., 2014
NSAIDs and analgesics	Naproxen	1,110	Vietnam	Tran et al., 2014
NSAIDs and analgesics	Acetaminophen	12,430	Nigeria	Ebele et al., 2020
NSAIDs and analgesics	Ibuprofen	2,740	Nigeria	Ebele et al., 2020
NSAIDs and analgesics	Naproxen	2,120	Nigeria	Ebele et al., 2020
NSAIDs and analgesics	Diclofenac	200	Nigeria	Ebele et al., 2020
NSAIDs and analgesics	Ibuprofen	121	Singapore	Wu et al., 2010
NSAIDs and analgesics	Diclofenac	38	Singapore	Wu et al., 2010
NSAIDs and analgesics	Naproxen	30	Singapore	Wu et al., 2010
NSAIDs and analgesics	Ibuprofen	34.9	Baltic Sea/Polish	Borecka et al., 2015
NSAIDs and analgesics	Naproxen	13,100	United States/California	Vidal-Dorsch et al., 2012
NSAIDs and analgesics	Ibuprofen	12,000	United States/California	Vidal-Dorsch et al., 2012
NSAIDs and analgesics	Acetaminophen	11,000	United States/California	Vidal-Dorsch et al., 2012
NSAIDs and analgesics	Diclofenac	180	United States/California	Vidal-Dorsch et al., 2012
NSAIDs and analgesics	Diclofenac	843	China	Yang et al., 2011
NSAIDs and analgesics	Ibuprofen	2,200	Taiwan	Fang et al., 2012
NSAIDs and analgesics	Diclofenac	185	Taiwan	Fang et al., 2012
NSAIDs and analgesics	Ketoprofen	184	Taiwan	Fang et al., 2012
NSAIDs and analgesics	Ibuprofen	143,000	Spain	Santos et al., 2007
NSAIDs and analgesics	Ketoprofen	2,100	Spain	Santos et al., 2007
NSAIDs and analgesics	Diclofenac	280	Spain	Santos et al., 2007
NSAIDs and analgesics	Ibuprofen	1,130	Japan	Nakada et al., 2006
NSAIDs and analgesics	Ketoprofen	369	Japan	Nakada et al., 2006
NSAIDs and analgesics	Ibuprofen	16,500	Canada	Lishman et al., 2006
NSAIDs and analgesics	Diclofenac	1,010	Canada	Lishman et al., 2006
NSAIDs and analgesics	Ketoprofen	289	Canada	Lishman et al., 2006
NSAIDs and analgesics	Ibuprofen	1,900	United States/Maryland	Yu et al., 2006
NSAIDs and analgesics	Ketoprofen	1,200	United States/Maryland	Yu et al., 2006
NSAIDs and analgesics	Diclofenac	110	United States/Maryland	Yu et al., 2006
NSAIDs and analgesics	Diclofenac	4,114	Austria	Clara et al., 2005
NSAIDs and analgesics	Ibuprofen	2,679	Austria	Clara et al., 2005
NSAIDs and analgesics	Ibuprofen	1,400	Switzerland	Tixier et al., 2003
NSAIDs and analgesics	Diclofenac	990	Switzerland	Tixier et al., 2003
NSAIDs and analgesics	Ketoprofen	180	Switzerland	Tixier et al., 2003
NSAIDs and analgesics	Ibuprofen	3,400	Germany	Ternes, 1998
NSAIDs and analgesics	Diclofenac	2,100	Germany	Ternes, 1998
NSAIDs and analgesics	Ketoprofen	380	Germany	Ternes, 1998
NSAIDs and analgesics	Ibuprofen	4,201	United Kingdom	Ashton et al., 2004
NSAIDs and analgesics	Diclofenac	599	United Kingdom	Ashton et al., 2004
Antibiotic	Azithromycin	597.5	Portugal	Rodriguez-Mozaz et al., 2020
Antibiotic	Ciprofloxacin	584.9	Portugal	Rodriguez-Mozaz et al., 2020
Antibiotic	Clarithromycin	313,2	Portugal	Rodriguez-Mozaz et al., 2020
Antibiotic	Tetracycline	231.2	Portugal	Rodriguez-Mozaz et al., 2020
Antibiotic	Trimethoprim	190.6	Portugal	Rodriguez-Mozaz et al., 2020
Antibiotic	Ofloxacin	184.9	Portugal	Rodriguez-Mozaz et al., 2020
Antibiotic	Clindamycin	86.6	Portugal	Rodriguez-Mozaz et al., 2020
Antibiotic	Sulfapyridine	48.8	Portugal	Rodriguez-Mozaz et al., 2020
Antibiotic	Cefalexin	38.4	Portugal	Rodriguez-Mozaz et al., 2020
Antibiotic	Sulfamethoxazole	30.2	Portugal	Rodriguez-Mozaz et al., 2020
Antibiotic	Pipemidic acid	20.1	Portugal	Rodriguez-Mozaz et al., 2020
Antibiotic	Azithromycin	299.5	Spain	Rodriguez-Mozaz et al., 2020
Antibiotic	Ciprofloxacin	200.3	Spain	Rodriguez-Mozaz et al., 2020
Antibiotic	Ofloxacin	142.3	Spain	Rodriguez-Mozaz et al., 2020
Antibiotic	Sulfamethoxazole	123.4	Spain	Rodriguez-Mozaz et al., 2020
Antibiotic	Clarithromycin	112	Spain	Rodriguez-Mozaz et al., 2020
Antibiotic	Trimethoprim	102.8	Spain	Rodriguez-Mozaz et al., 2020
Antibiotic	Clindamycin	101.4	Spain	Rodriguez-Mozaz et al., 2020
Antibiotic	Metronidazole	76.1	Spain	Rodriguez-Mozaz et al., 2020
Antibiotic	Enrofloxacin	69.4	Spain	Rodriguez-Mozaz et al., 2020
Antibiotic	Cefalexin	65.2	Spain	Rodriguez-Mozaz et al., 2020
Antibiotic	Sulfapyridine	63.9	Spain	Rodriguez-Mozaz et al., 2020
Antibiotic	Pipemidic acid	30.1	Spain	Rodriguez-Mozaz et al., 2020
Antibiotic	Ciprofloxacin	316.8	Cyprus	Rodriguez-Mozaz et al., 2020
Antibiotic	Ofloxacin	305.1	Cyprus	Rodriguez-Mozaz et al., 2020
Antibiotic	Trimethoprim	74.2	Cyprus	Rodriguez-Mozaz et al., 2020
Antibiotic	Sulfamethoxazole	68.5	Cyprus	Rodriguez-Mozaz et al., 2020
Antibiotic	Cefalexin	66.3	Cyprus	Rodriguez-Mozaz et al., 2020
Antibiotic	Sulfapyridine	48.7	Cyprus	Rodriguez-Mozaz et al., 2020
Antibiotic	Azithromycin	48	Cyprus	Rodriguez-Mozaz et al., 2020
Antibiotic	Tetracycline	36.9	Cyprus	Rodriguez-Mozaz et al., 2020
Antibiotic	Clindamycin	27.8	Cyprus	Rodriguez-Mozaz et al., 2020
Antibiotic	Metronidazole	19.6	Cyprus	Rodriguez-Mozaz et al., 2020
Antibiotic	Pipemidic acid	15.2	Cyprus	Rodriguez-Mozaz et al., 2020
Antibiotic	Clarithromycin	11.9	Cyprus	Rodriguez-Mozaz et al., 2020
Antibiotic	Orbifloxacin	6.7	Cyprus	Rodriguez-Mozaz et al., 2020
Antibiotic	Azithromycin	266.7	Ireland	Rodriguez-Mozaz et al., 2020
Antibiotic	Ciprofloxacin	259.8	Ireland	Rodriguez-Mozaz et al., 2020
Antibiotic	Clarithromycin	204.4	Ireland	Rodriguez-Mozaz et al., 2020
Antibiotic	Tetracycline	194.2	Ireland	Rodriguez-Mozaz et al., 2020
Antibiotic	Trimethoprim	141.3	Ireland	Rodriguez-Mozaz et al., 2020
Antibiotic	Ampicillin	99.4	Ireland	Rodriguez-Mozaz et al., 2020
Antibiotic	Sulfapyridine	95.5	Ireland	Rodriguez-Mozaz et al., 2020
Antibiotic	Metronidazole	88.6	Ireland	Rodriguez-Mozaz et al., 2020
Antibiotic	Cefalexin	87.6	Ireland	Rodriguez-Mozaz et al., 2020
Antibiotic	Ofloxacin	65.4	Ireland	Rodriguez-Mozaz et al., 2020
Antibiotic	Clindamycin	59.1	Ireland	Rodriguez-Mozaz et al., 2020
Antibiotic	Sulfamethoxazole	53	Ireland	Rodriguez-Mozaz et al., 2020
Antibiotic	Nalidixic acid	50.3	Ireland	Rodriguez-Mozaz et al., 2020
Antibiotic	Pipemidic acid	18.2	Ireland	Rodriguez-Mozaz et al., 2020
Antibiotic	Oxolinic Acid	5.3	Ireland	Rodriguez-Mozaz et al., 2020
Antibiotic	Azithromycin	290.4	Germany	Rodriguez-Mozaz et al., 2020
Antibiotic	Ciprofloxacin	230.6	Germany	Rodriguez-Mozaz et al., 2020
Antibiotic	Clarithromycin	123.4	Germany	Rodriguez-Mozaz et al., 2020
Antibiotic	Sulfapyridine	112	Germany	Rodriguez-Mozaz et al., 2020
Antibiotic	Clindamycin	110.7	Germany	Rodriguez-Mozaz et al., 2020
Antibiotic	Trimethoprim	105	Germany	Rodriguez-Mozaz et al., 2020
Antibiotic	Ofloxacin	66.5	Germany	Rodriguez-Mozaz et al., 2020
Antibiotic	Sulfamethoxazole	34.9	Germany	Rodriguez-Mozaz et al., 2020
Antibiotic	Metronidazole	20.3	Germany	Rodriguez-Mozaz et al., 2020
Antibiotic	Tetracycline	15.4	Germany	Rodriguez-Mozaz et al., 2020
Antibiotic	Pipemidic acid	11.8	Germany	Rodriguez-Mozaz et al., 2020
Antibiotic	Cefalexin	308	Finland	Rodriguez-Mozaz et al., 2020
Antibiotic	Trimethoprim	186.7	Finland	Rodriguez-Mozaz et al., 2020
Antibiotic	Azithromycin	130.7	Finland	Rodriguez-Mozaz et al., 2020
Antibiotic	Sulfapyridine	98.8	Finland	Rodriguez-Mozaz et al., 2020
Antibiotic	Clindamycin	94.2	Finland	Rodriguez-Mozaz et al., 2020
Antibiotic	Tetracycline	70.6	Finland	Rodriguez-Mozaz et al., 2020
Antibiotic	Ciprofloxacin	43.2	Finland	Rodriguez-Mozaz et al., 2020
Antibiotic	Metronidazole	41.9	Finland	Rodriguez-Mozaz et al., 2020
Antibiotic	Ofloxacin	22.8	Finland	Rodriguez-Mozaz et al., 2020
Antibiotic	Clarithromycin	4.8	Finland	Rodriguez-Mozaz et al., 2020
Antibiotic	Pipemidic acid	4.8	Finland	Rodriguez-Mozaz et al., 2020
Antibiotic	Sulfapyridine	184	Norway	Rodriguez-Mozaz et al., 2020
Antibiotic	Tetracycline	179.2	Norway	Rodriguez-Mozaz et al., 2020
Antibiotic	Ciprofloxacin	159.2	Norway	Rodriguez-Mozaz et al., 2020
Antibiotic	Azithromycin	149.7	Norway	Rodriguez-Mozaz et al., 2020
Antibiotic	Trimethoprim	119.7	Norway	Rodriguez-Mozaz et al., 2020
Antibiotic	Clindamycin	97.1	Norway	Rodriguez-Mozaz et al., 2020
Antibiotic	Metronidazole	93.2	Norway	Rodriguez-Mozaz et al., 2020
Antibiotic	Cefalexin	60.7	Norway	Rodriguez-Mozaz et al., 2020
Antibiotic	Sulfamethoxazole	48.6	Norway	Rodriguez-Mozaz et al., 2020
Antibiotic	Ofloxacin	27.1	Norway	Rodriguez-Mozaz et al., 2020
Antibiotic	Clarithromycin	20.8	Norway	Rodriguez-Mozaz et al., 2020
Antibiotic	Pipemidic acid	7,5	Norway	Rodriguez-Mozaz et al., 2020
Antibiotic	Oxytetracycline	2,796.6	China	Wang et al., 2017
Antibiotic	Tetracycline	1,454.8	China	Wang et al., 2017
Antibiotic	Chlorotetracycline	876.2	China	Wang et al., 2017
Antibiotic	Sulfamethoxazole	715.3	China	Wang et al., 2017
Antibiotic	Sulfadiazine	499.5	China	Wang et al., 2017
Antibiotic	Sulfamerazine	329.1	China	Wang et al., 2017
Antibiotic	Fleroxacin	309.4	China	Wang et al., 2017
Antibiotic	Difloxacin	250.2	China	Wang et al., 2017
Antibiotic	Sulfanomethioxine	225.5	China	Wang et al., 2017
Antibiotic	Ofloxazin	203.7	China	Wang et al., 2017
Antibiotic	Sulfadiamidine	109.9	China	Wang et al., 2017
Antibiotic	Ciprofloxacin	106.2	China	Wang et al., 2017
Antibiotic	Sulfameter	6	China	Wang et al., 2017
Antibiotic	Sulfamethoxazole	2,010	Mexico	Rivera-Jaimes et al., 2018
Antibiotic	Trimethoprim	790	Mexico	Rivera-Jaimes et al., 2018
Antibiotic	Erythromycin	160	South Africa	Matongo et al., 2015
Antibiotic	Ciprofloxacin	14,300	South Africa	Agunbiade and Moodley, 2016
Antibiotic	Sulfaguanidine	46,000	South Africa	Madikizela et al., 2020
Antibiotic	Spiramycin	38,200	South Africa	Madikizela et al., 2020
Antibiotic	Fluoroquinolones	900	South Africa	Hendricks and Pool, 2012
Antibiotic	Ciprofloxacin	1,360	South Africa	Agunbiade and Moodley, 2016
Antibiotic	Erythromycin	10,600	Ghana	Azanu et al., 2018
Antibiotic	Sulfamethoxazole	3,600	Ghana	Azanu et al., 2018
Antibiotic	Metronidazole	363	Ghana	Azanu et al., 2018
Antibiotic	Ciprofloxacin	15,730	Ghana	Azanu et al., 2018
Antibiotic	Erythromycin	16,400	Tunisia	Tahrani et al., 2017
Antibiotic	Ofloxacin	175	Tunisia	Harrabi et al., 2018
Antibiotic	Enrofloxacin	400	Tunisia	Harrabi et al., 2018
Antibiotic	Trimethoprim	7,800	Tunisia	Tahrani et al., 2017
Antibiotic	Sulfamethoxazole	53,800	Mozambique	Branchet et al., 2019
Antibiotic	Trimethoprim	11,400	Mozambique	Segura et al., 2015
Antibiotic	Sulfamethoxazole	23,300	Kenya	K’oreje et al., 2012
Antibiotic	Sulfadoxin	1,040	Kenya	K’oreje et al., 2018
Antibiotic	Doxycycline	32,200	Kenya	Kairigo et al., 2020
Antibiotic	Norfloxacin	26,600	Kenya	Kairigo et al., 2020
Antibiotic	Trimethoprim	94,800	Kenya	K’oreje et al., 2012
Antibiotic	Sulfamethoxazole	5,600	Uganda	Nantaba et al., 2020
Antibiotic	Trimethoprim	89	Uganda	Nantaba et al., 2020
Antibiotic	Enrofloxacin	440	Nigeria	Olaitan et al., 2017
Antibiotic	Oxytetracycline	26	Nigeria	Olaitan et al., 2017
Antibiotic	Cefuroxime	868	Nigeria	Olaitan et al., 2017
Antibiotic	Amoxicillin	272,200	Nigeria	Ebele et al., 2020
Endocrine disruptors	Di-(2-ethylhexyl) phthalate	589	Australia	Tan et al., 2007
Endocrine disruptors	nonylphenol	335	Australia	Tan et al., 2007
Endocrine disruptors	Dibutyl phthalate	101	Australia	Tan et al., 2007
Endocrine disruptors	Bisphenol A	86.7	Australia	Tan et al., 2007
Endocrine disruptors	Benzyl butyl phthalate	75.7	Australia	Tan et al., 2007
Endocrine disruptors	Diethyl phthalate	36.9	Australia	Tan et al., 2007
Endocrine disruptors	4-tert-octylphenol	23.5	Australia	Tan et al., 2007
Endocrine disruptors	4-cumylphenol	1.9	Australia	Tan et al., 2007
Antiretroviral	Efavirenz	37.3	South Africa	Mlunguza et al., 2020
Antiretroviral	Emtricitabine	1.47	South Africa	Mlunguza et al., 2020
Antiretroviral	Tenofovir disproxil	0.25	South Africa	Mlunguza et al., 2020
Antiretroviral	Lamvudine	118,970	Zambia	Ngumba et al., 2020
Antiretroviral	Zidovudine	66,590	Zambia	Ngumba et al., 2020
Antiretroviral	Nevirapine	1,720	Zambia	Ngumba et al., 2020
Antiretroviral	Nevirapine	33,440	Kenya	K’oreje et al., 2012
Antiretroviral	Zidovudine	18,300	Kenya	K’oreje et al., 2012
Antiretroviral	Lamvudine	3,150	Kenya	K’oreje et al., 2012
Antiretroviral	Valacyclovir	21	Japan	Azuma et al., 2019
Antiretroviral	Zidovudine	564	Germany	Prasse et al., 2010
Antiretroviral	Nevirapine	32.1	Germany	Boulard et al., 2018
Antiretroviral	Abacavir	10	Germany	Boulard et al., 2018
Antiretroviral	Darunavir	169	Poland	Giebułtowicz et al., 2018
Antiretroviral	Zidovudine	191	France	Aminot et al., 2015
Antiretroviral	Ritonavir	155	France	Aminot et al., 2015
Antiretroviral	Lamivudine	44	France	Aminot et al., 2015
Antiretroviral	Nevirapine	7.7	France	Aminot et al., 2015
Antiretroviral	Indinavir	1.5	France	Aminot et al., 2015
Antiretroviral	Saquinavir	0.2	France	Aminot et al., 2015
Antiretroviral	Lamivudine	507	Belgium	Vergeynst et al., 2015
Antiretroviral	Ritonavir	108	Switzerland	Kovalova et al., 2012
Antiretroviral	Lamivudine	355	United States	Masoner et al., 2014
Antiretroviral	Abacavir	185	United States	Masoner et al., 2014
Antiretroviral	Nevirapine	25.2	United States	Fisher et al., 2016
Anticancer	Capecitabine	46	Portugal	Cristóvão et al., 2021
Anticancer	Ifosamide	44	Portugal	Cristóvão et al., 2021
Anticancer	Cyclophosphamide	17	Portugal	Cristóvão et al., 2021
Anticancer	Tamoxifen	181	Spain	Negreira et al., 2014
Anticancer	Cytarabine	924	Canada	Vaudreuil et al., 2020
Anticancer	Difluorodeoxyuridine	300	Canada	Vaudreuil et al., 2020
Anticancer	Cyclophosphamide	118	Canada	Vaudreuil et al., 2020
Anticancer	Methotrexate	27.3	Canada	Vaudreuil et al., 2020

Non-steroidal anti-inflammatory drugs and analgesics are some of the most important groups of pharmaceutical products worldwide, with diverse chemical structures and similar therapeutic effects, having an estimated annual production of several hundred tons (Comber et al., 2018). Large amounts of anti-inflammatory drugs are prescribed in human care, but they are often sold in much higher amounts without a prescription (Ternes, 2001). NSAIDs and analgesics are often combined with antibiotics in veterinary medicine for problems such as pain, inflammation, fever, osteoarthritis and arthritis, and to reduce stress (Courtheyn et al., 2002; Bártíková et al., 2016). However, these two types of pharmaceuticals have numerous adverse effects in humans, including gastrointestinal disturbances, ulceration, renal failure with increased risk of post-operative bleeding, asthma, and rare allergic reactions (Ben Maamar et al., 2017; Morelli et al., 2017; Borgeat et al., 2018; Hurtado-Gonzalez et al., 2021). Approximately 35 million people use NSAIDs every day worldwide (Yu et al., 2013), and China increased its domestic production from 41,537 t in 2013 to 46,673 t in 2017 (Yan et al., 2021). They are currently monitored in effluents worldwide to check these drug concentrations and several studies show that both NSAIDs and analgesics are commonly detected in water bodies (Balakrishna et al., 2017; Świacka et al., 2021). In Cuernavaca (Mexico), high concentrations of naproxen (732–4,889 ng/L), acetaminophen (354–4,460 ng/L), and diclofenac (258–1,398 ng/L) have been detected in samples collected in different years, in the influent and effluent of a WWTP and in the surface waters of the Apatalco River (Rivera-Jaimes et al., 2018). Furthermore, the drugs diclofenac (10,221 ng/L highest concentration detected) and acetaminophen (1234-2346 ng/L), among others, have been detected in effluents from the Red Sea (Saudi Arabia) (Ali et al., 2017). On the other hand, in Brazil, acetaminophen (17.4–34.6 ng/L), diclofenac (19.4 ng/L), and ibuprofen (326.1–2,094.4 ng/L) have been detected in the surface and bottom water samples from Santos Bay (Pereira et al., 2016). These same drugs have also been detected in surface water on the northern Antarctic Peninsula region due to increased tourism in this area, with concentrations of 48.74, 15.09, and 10.05 ng/L of acetaminophen, diclofenac, and ibuprofen, reported respectively (González-Alonso et al., 2017).

Among the pharmaceutical compounds found in wastewater, antibiotics are of the greatest concern due to their persistent nature, partial metabolism, and easy movement through ecosystems (Mukhtar et al., 2020). Antibiotic production in China was approximately 92,700 tons, 48% destined for humans and the remaining for livestock; a total of 46% active metabolites were produced (Zafar et al., 2021). The antibiotics most commonly found in wastewater are sulfonamides, quinolones, tetracyclines, fluoroquinolones, and nitroimidazoles. The total concentrations of antibiotics vary depending on the body of water, in the case of wastewater, they can range between 0.0013 and 0.0125 μg/mL, in drinking water 0.0005 and 0.0214 μg/mL and river water 0.0003 and 0.0039 μg/mL (Zhang et al., 2015; Pan and Chu, 2017; Hanna et al., 2018). Antibiotic resistance of microorganisms to antimicrobials is becoming even stronger and more widespread over time and is expected to greatly increase human morbility and mortality in the near future (Bondarczuk and Piotrowska-Seget, 2019). Antibiotics have been found in rivers all over the world, including several in Spain (Ebro, Guadarrama and Manzanares Rivers), Italy (Arno River), South Korea (Han River), Taiwan (Xindian, Gaoping, Dahan and Po River), France (Seine River), United States (Ozark River), Sweden (Hoje River), and China (Pearl, Hai, Liao and Yellow Rivers) (Peng et al., 2008, 2011; Valcárcel et al., 2011; López-Serna et al., 2013; Bilal et al., 2020).

Endocrine disruptors were defined in 2002 by the International Programme on Chemical Safety (IPCS) of the United Nations Environment Programme (UNEP) and by the World Health Organization (WHO) as “an exogenous substance or mixture that alters the function(s) of the endocrine system and consequently causes adverse health effects in an intact organism or population”. Among the most common endocrine disruptors are pesticides, bisphenols and natural hormones (Gore et al., 2014; Tijani et al., 2016). These substances are not removed from water by conventional treatment processes and are found in wastewater bodies in the order of nanograms to micrograms per liter (Andrade-Eiroa et al., 2016; Gröger et al., 2020; Li et al., 2020).

Antiretroviral drugs are frequently used to treat the human immunodeficiency virus (HIV), an epidemic that has developed worldwide and has its epicenter in South Africa (Tompsett, 2020). As a result, millions of people have access to these drugs on a daily basis, with more than 40 different antiretroviral drugs being used for the treatment of HIV. These include abacavir, efavirenz, lamivudine, nevirapine, tenofovir, and zidovudine; many of which are used in combination (Russo et al., 2018; Mlunguza et al., 2020). As a consequence of the increase in the rate of HIV infection over the years, there has been a significant increase in the production and consumption of antiretroviral drugs worldwide (Nannou et al., 2020; Reddy et al., 2021). In addition, as consequence of the new pandemic coronavirus (COVID-19), antiretroviral drugs have also been used for the treatment of SARS-CoV-2. In some countries, such as China and Japan, clinical trials have been conducted to test the efficiency of using HIV drugs to treat COVID-19 (Reddy et al., 2021). At the moment, a scarcity of studies has dealt with this new issue. However, some studies have started to show a relevant problem that we will have in the very near future (Mupatsi, 2020).

In the coming decades, annual cancer cases are expected to increase to more than 20 million, which means an exponential increase in anticancer drugs and their subsequent release into wastewater (Ferlay et al., 2013). Most of these compounds are incompletely assimilated and metabolized by the human body, thus excreted in feces and urine. The most commonly administered anticancer drugs include cyclophosphamide, tamoxifen, ifosfamide and methotrexate, among others. These drugs have been detected in surface water, WWTP effluents and influents, and hospital effluents. Detected concentrations of cyclophosphamide range from 0.05 to 22,100 ng/L, ifosfamide 0.14–86,200 ng/L, methotrexate 1.6–4,756 ng/L, and tamoxifen 0.01–740 ng/L (Nassour et al., 2020). Several studies have detected these drugs in water masses, confirming that current water treatment systems fail to degrade them (Verlicchi et al., 2010; Cristóvão et al., 2019). Different international agencies have developed protocols for the handling and storing of pharmaceuticals to reduce their harmful effect on the environment (Bernabeu-Martínez et al., 2018). One of the main concerns is that these drugs may suffer biomagnification (Yadav et al., 2021).

## Impact of Pharmaceuticals on the Environment and Living Organisms

Since almost all drugs are not completely metabolized by organisms (usually a small fraction of the active site of drug metabolic enzymes are occupied, the half-life of drugs are limited, and drugs are administrated in higher amounts than necessary to increase efficiency) (Coleman, 2020), the compounds that can cause the most damage once they are excreted and reached wastewater are PhACs. They are also called active pharmaceutical ingredients or APIs and metabolites, referring to the molecules resulting from these original compounds due to structural changes that take place in organisms. In addition, the resulting molecules are also subject to changes in the environment (such as oxidation, photolysis, or biotransformation). These changes can occur through both biotic and abiotic processes. Thus, many pharmaceutical products are biotransformed by microorganisms (Kümmerer, 2009; Wu et al., 2012). Ecotoxicologists are increasingly concerned about the worldwide detection of pharmaceutical residues in aquatic environments since their long-term toxic effects are being increasingly studied. However, it is challenging to know these effects because of the short time period these substances have been present in the environment (Nantaba et al., 2020; Ramírez-Morales et al., 2020; Gani et al., 2021).

Different studies analyzed the microbiome of wastewater where, in the case of hospitals, an abundance of anaerobes related to pathogenic threats such as Bifidobacteriales, Bacteroidales, and Clostridiales was found (Buelow et al., 2018; Ogwugwa et al., 2021; Palanisamy et al., 2021). They also noted that compared to other locations, hospital wastewater contains microorganisms with higher relative levels of antimicrobial and antibiotic resistance genes (Buelow et al., 2018). The mycobiome of hospital wastewater has also been analyzed, indicating the presence of different opportunistic phyla such as *Mycosphaerella*, *Drechslera*, *Candida*, or *Cyphellophora* (Olicón-Hernández et al., 2021), whose risk that they may acquire resistance to antibiotics is of great concern and may have great repercussions for global health.

### Beta-Blocker and Psychoactives

β-blockers are a group of pharmaceuticals that are commonly detected in the environment. This is because many wastewater plants are not adapted to remove these micropollutants. Detected concentrations vary from 3 to 6,167 ng/L, which are already sufficient to cause neurotoxic and reproductive disorders in living organisms (Godlewska et al., 2021). Bisoprolol causes immobilization in *Daphnia similis* (Godoy et al., 2019) and mortality in fish and green algae (Fonseca et al., 2021). Propranolol causes growth and development problems in algae such as *Synechococcus leopolensis* and *Cyclotella meneghiniana* (Ferrari et al., 2004), mortality in crustacea (*Ceriodaphnia dubia*) (Huggett et al., 2002), and embryonic development problems in *Danio rerio* (Bittner et al., 2018).

Psychoactive substances affect thought, emotion, will and behavior (Jin et al., 2022). According to their pharmacological properties, psychoactive substances (including legal and illegal drugs) are opioids, cannabis, central nervous system depressants, central nervous system stimulants, hallucinogens, and tobacco (Schlüsener et al., 2015; Tanoue et al., 2019). These substances have different effects on humans, such as analgesia, anesthesia, inability to concentrate, excitement, anxiety, and mania. Jin et al. (2022) indicated that ecological risk assessment is a crucial part of research on psychoactive substances, as the current relevant literature is scarce. Due to the biological activity of such substances, there is a need for rapid improvement of risk assessment, including acute, cone and developmental toxicity, neurotoxicity, and endocrine-disrupting effects, among others, as well as the development of remediation technologies.

### Non-steroidal Anti-inflammatory Drugs and Analgesics

Pharmaceuticals are known to have biological effects on living organisms, but there is not enough information currently available to assess the possible ecotoxicological impacts. Below are some of the toxic and ecological risks of NSAIDs and analgesics, according to various studies and summarized in Table 2: (I) population declines of *Gyps* vultures in Asia due to high diclofenac concentration (Cuthbert et al., 2007); (II) diclofenac impairs prostate gland synthesis and damage to the gills, liver, and kidneys of *Salmo trutta f. fario* (Hoeger et al., 2005); (III) histological alterations of the kidneys and gills, cytological alterations of the liver, kidneys, and gills, and deterioration of ionic regulation in *Oncorhynchus mykiss* (Schwaiger et al., 2004; Triebskorn et al., 2004; Gravel et al., 2009); (IV) ibuprofen, diclofenac, naproxen and ketoprofen inhibits CYP2M in *Cyprinus carpio* (Thibaut et al., 2006); (V) ibuprofen change breeding pattern of *Oryzias latipes* (Flippin et al., 2007); (VI) ibuprofen, diclofenac, and acetaminophen cause cardiovascular abnormalities, hatch and motor behavior and interruption of oocyte maturation/ovulation in *D. rerio* (David and Pancharatna, 2009; Lister and Van Der Kraak, 2009; Xia et al., 2017); (VII) diclofenac alters estrogenic activity, response of specific tissue biomarkers, decreased superoxide dismutase, and glutathione reductase activities in gills, and high catalase activity and levels of lipid peroxidation in the digestive gland in *Mytilus galloprovincialis* (Gonzalez-Rey and Bebianno, 2014). As can be inferred, high concentrations of NSAIDs and analgesics in the environment, such as acetylsalicylic acid, acetaminophen, diclofenac, ibuprofen, and naproxen, cause serious environmental problems (Parolini, 2020). In addition to fish, the main organisms affected are invertebrates, including arthropods, mollusks, cnidarians and rotifers (Parolini, 2020). NSAIDs also affect the plant growth of species such as *Pisum sativum* and *Vigna unguiculata* (Svobodníková et al., 2020; Wijaya et al., 2020; Table 2).

**TABLE 2:** Impact of pharmaceuticals on the environment and humans.

Pharmaceutical type	Impact	References
β-blockers (bisoprolol)	Inmobilization in *Daphnia similis*	Godoy et al., 2019
β-blockers (bisoprolol)	Mortality in green algae	Fonseca et al., 2021
β-blockers (bisoprolol)	Mortality in fish	Fonseca et al., 2021
β-blockers (propanolol)	Growth and development problems in algae such as *Synechococcus leopolensis* and *Cyclotella meneghiniana*	Ferrari et al., 2004
β-blockers (propanolol)	Mortality in crustacea (*Ceriodaphnia dubia*)	Huggett et al., 2002
β-blockers (propanolol)	Embryonic development problems in *Danio rerio*	Bittner et al., 2018
NSAIDs and analgesics (Acetaminophen)	Cardiovascular abnormalities, hatch and motor behavior and interruption of oocyte maturation/ovulation in *Danio rerio*	David and Pancharatna, 2009; Lister and Van Der Kraak, 2009; Xia et al., 2017
NSAIDs and analgesics (Diclofenac)	Population declines of Gyps vultures	Cuthbert et al., 2007
NSAIDs and analgesics (Diclofenac)	Prostate gland synthesis and damage to the gills, liver, and kidneys of *Salmo trutta f. fario*	Hoeger et al., 2005
NSAIDs and analgesics (Diclofenac)	Histological alterations of the kidneys and gills, cytological alterations of the liver, kidneys, and gills, and deterioration of ionic regulation in *Oncorhynchus mykiss*	Schwaiger et al., 2004; Triebskorn et al., 2004; Gravel et al., 2009
NSAIDs and analgesics (Diclofenac)	Inhibits CYP2M in *Cyprinus carpio*	Thibaut et al., 2006
NSAIDs and analgesics (Diclofenac)	Cardiovascular abnormalities, hatch and motor behavior and interruption of oocyte maturation/ovulation in *Danio rerio*	David and Pancharatna, 2009; Lister and Van Der Kraak, 2009; Xia et al., 2017
NSAIDs and analgesics (Diclofenac)	Alteration of estrogenic activity, response of specific tissue biomarkers, decreased superoxide dismutase and glutathione reductase activities in gills, and high catalase activity and levels of lipid peroxidation in the digestive gland in *Mytilus galloprovincialis*	Gonzalez-Rey and Bebianno, 2014
NSAIDs and analgesics (Ibuprofen)	Inhibits CYP2M in *Cyprinus carpio*	Thibaut et al., 2006
NSAIDs and analgesics (Ibuprofen)	Change breeding pattern of *Oryzias latipes*	Flippin et al., 2007
NSAIDs and analgesics (Ibuprofen)	Cardiovascular abnormalities, hatch and motor behavior and interruption of oocyte maturation/ovulation in *Danio rerio*	David and Pancharatna, 2009; Lister and Van Der Kraak, 2009; Xia et al., 2017
NSAIDs and analgesics (Ibuprofen)	Reduce the shoot and root lengths, fresh and dry weights, leaf area, and chlorophyll a and b, carotenoid, total chlorophyll, mineral (K and Mg), glutathione reductase, and soluble protein contents of *Vigna unguiculata*	Wijaya et al., 2020
NSAIDs and analgesics (Ketoprofen)	Inhibits CYP2M in *Cyprinus carpio*	Thibaut et al., 2006
NSAIDs and analgesics (Naproxen)	Inhibits CYP2M in *Cyprinus carpio*	Thibaut et al., 2006
NSAIDs and analgesics (Naproxen)	*Pisum sativum*	Svobodníková et al., 2020
Antibiotics	Algae and aquatic plants are severely affected	Brain et al., 2008; Brausch et al., 2012
Antibiotics	Block the electron chain of photosystems II and increase oxidative stress (photosynthesis inhibitors)	Nie et al., 2013
Antibiotics	Bacteria seem to be developing resistance to antibacterial substances due to exposure to low concentrations over several generations	Kollef et al., 2017; Willyard, 2017; García et al., 2020; Wang et al., 2020;
Antibiotics	*Hydra attenuata* show relatively low toxicity	Wollenberger et al., 2000; Kołodziejska et al., 2013; Minguez et al., 2016
Antibiotics	Crustaceans such as *Artemia salina*, *Daphnia magna*, and *Ceriodaphnia dubia* show relatively low acute toxicity	Wollenberger et al., 2000; Kołodziejska et al., 2013; Minguez et al., 2016
Antibiotics	Invertebrates such as *Hydra attenuata* and crustaceans such as *Artemia salina*, *Daphnia magna*, and *Ceriodaphnia dubia* show relatively low acute toxicity in the presence of antibiotics	Wollenberger et al., 2000; Kołodziejska et al., 2013; Minguez et al., 2016
Endocrine disruptors	Block or imitate the natural hormones responsible for the functioning of some organs, in both humans and animals	Vieira et al., 2020
Endocrine disruptors	Alter the reproductive system	Heindel et al., 2015; Braun, 2017; Nadal et al., 2017
Endocrine disruptors	Cause Alzheimer’s disease	Heindel et al., 2015; Braun, 2017; Nadal et al., 2017
Endocrine disruptors	Thyroid problems	Heindel et al., 2015; Braun, 2017; Nadal et al., 2017
Endocrine disruptors	Obesity and/or cancer	Heindel et al., 2015; Braun, 2017; Nadal et al., 2017
Endocrine disruptors	Affected the reproductive system	Vieira et al., 2020
Endocrine disruptors	Levels of vitellogenin and hatchability	Vieira et al., 2020
Anticancer drugs	Cytotoxic, genotoxic, mutagenic, and teratogenic effects in any eukaryotic organism	Kümmerer et al., 2000; Johnson et al., 2008
Anticancer drugs	Groups at greatest risk are children, pregnant women, and the elderly	Rowney et al., 2009
Anticancer drugs	Caused histopathological changes in the liver and kidney and impaired the integrity of their DNA, introducing massive changes in the entire transcriptome in *Danio rerio*	Kovács et al., 2015; Gajski et al., 2016
Antiretroviral drugs	Resistant strains of HIV can be created in the body through exposure to water contaminated with these drugs	Daouk et al., 2015; Ncube et al., 2018
Antiretroviral drugs	Anemia	Ncube et al., 2018
Antiretroviral drugs	Nausea	Ncube et al., 2018
Antiretroviral drugs	Hypersensitivity	Ncube et al., 2018
Antiretroviral drugs	Nephrotoxicity and renal failure	Ncube et al., 2018
Antiretroviral drugs	Rash	Ncube et al., 2018

### Antibiotics

Due to the continuous introduction of antibiotics into the environment, aquatic and soil organisms are chronically exposed to these drugs (Gothwal and Shashidhar, 2015; Bengtsson-Palme and Larsson, 2016). Moreover, because they are active at very low concentrations, they have a toxic effect on organisms, and there is a synergistic effect when they are present together with other drugs and/or xenobiotic compounds (González-Pleiter et al., 2013). Algae and aquatic plants are severely affected by antibiotics (Brain et al., 2008; Brausch et al., 2012). Many of them have been found to be photosynthesis inhibitors, as they can block the electron chain of photosystems II and increase oxidative stress (Nie et al., 2013). However, microorganisms, including bacteria and fungi, are developing resistance to antibacterial substances due to exposure to low concentrations over several generations (Kollef et al., 2017; Willyard, 2017; García et al., 2020; Wang et al., 2020). Invertebrates such as *Hydra attenuata* and crustaceans such as *Artemia salina*, *Daphnia magna*, and *Ceriodaphnia dubia* show relatively low acute toxicity in the presence of antibiotics (Wollenberger et al., 2000; Kołodziejska et al., 2013; Minguez et al., 2016). On the other hand, in fish, acute toxicity was only found at high concentrations, but there were cases in which no toxicity was observed (Santos et al., 2010; Brausch et al., 2012; Minguez et al., 2016; Table 2). The other major problem is antibiotic resistance genes (ARGs), which are genes that confer antibiotic resistance to bacteria, and can proliferate through the reproduction of antibiotic-resistant bacteria from the host or through horizontal gene transfer, are present in the environment, and thus considered as emerging environmental contaminants (Nadimpalli et al., 2020; Hu et al., 2021). Although treated wastewater contains significantly lower amounts of ARGs than untreated wastewater, several studies show that aquatic environments downstream of treatment plants can increase the amounts of ARGs because they are carried by mobile genetic elements, such as conjugative plasmids, integrative and conjugative elements, and transposons and integrons (Amos et al., 2018; Freeman et al., 2018; Jäger et al., 2018; Karkman et al., 2018; Liu et al., 2018). These effective carriers of ARGs could confer multi-resistance. One of the most detected genetic components in both effluents and aquatic environments is Class 1 integron-integrase gene (*intI1*) associated more frequently with ARGs and involved in horizontal gene transfer (Gillings et al., 2015; Cacace et al., 2019).

### Endocrine Disruptors

Endocrine disruptors seriously affect both human and animal health, as they act directly on the endocrine system and block or mimic the natural hormones responsible for the functioning of some organs (Vieira et al., 2020). These substances have been studied extensively in humans, nevertheless, much less in the environment. It is known that they can alter the reproductive system, cause Alzheimer’s disease, thyroid problems, obesity and/or cancer (prostate, breast or endometrium cancer), among others (Heindel et al., 2015; Forte et al., 2016, 2019; Braun, 2017; Nadal et al., 2017; Marotta et al., 2019). In natural ecosystems, the reproductive system is also affected, as well as the levels of vitellogenin and hatchability and thus feminization with the consequent threat to the preservation of biodiversity (Vieira et al., 2020; Akhbarizadeh et al., 2021; Table 2).

### Antiretrovirals

In contrast to other pharmaceuticals, antiretrovirals, despite being abundant in wastewater, are poorly monitored, although some studies report on them (Ngumba et al., 2016; Abafe et al., 2018; Rimayi et al., 2018; Mosekiemang et al., 2019; Mtolo et al., 2019). These drugs could pass through treated wastewater in WWTPs, reach drinking water sources, and cause serious ecotoxicological problems for human health (Hawkins, 2010; Ncube et al., 2018; Mlunguza et al., 2020). Currently, the greatest concern is that resistant strains of HIV can be created in the body through exposure to water contaminated with these drugs (Daouk et al., 2015; Ncube et al., 2018; Table 2).

### Anticancer Drugs

Although anticancer drugs are designed to eliminate fast-growing cells, such as tumor cells, many of these drugs are not selective (Chari, 2008). This means that in addition to attacking healthy cells, they can cause cytotoxic, genotoxic, mutagenic, and teratogenic effects, i.e., cause adverse effects in any eukaryotic organism (Kümmerer et al., 2000; Johnson et al., 2008). For this reason, anticancer drugs are considered to be of great environmental concern, and especially the groups at greatest risk are children, pregnant women, and the elderly (Rowney et al., 2009). It has been shown that chronic exposure of two generations of *D. rerio* to anticancer drugs caused histopathological changes in the liver and kidney and impaired the integrity of their DNA, introducing massive changes in the entire transcriptome (Kovács et al., 2015; Gajski et al., 2016; Table 2).

Residues of pharmaceuticals in the environment typically occur as complex mixtures and even if the concentrations of an individual compound are low, the “cocktail effect” could be of significant ecotoxicological importance (Heath et al., 2016). To date, many works have focused on the study of individual organisms and analyzed a single drug or several drugs as a whole, but there are no works studying the impact of drugs on several populations simultaneously. This would provide essential information on ecotoxicity and the “domino effect” that affects individuals in a trophic chain since, in addition to bioaccumulation, the chain could be broken because a drug lethally affects a group of individuals.

## Development of Bioremediation Technologies

Improving technologies for drug elimination from wastewater is an important task since pharmaceuticals have been detected in effluent from WWTPs and consequently surface water, groundwater, and drinking water globally (Bartolo et al., 2021). Although the pharmaceuticals are found in concentrations ranging from the nanogram to microgram per liter, which is too low to cause acute toxicity, they are biologically active compounds that have the potential for chronic toxicity, bioaccumulation, and biomagnification (Ruan et al., 2020). Additionally, microplastics have been shown to serve as vectors for pharmaceuticals (Santos et al., 2021), thus increasing the exposure potential. Because of incomplete elimination during conventional wastewater treatment (Reyes et al., 2021) and the potential risk posed to the environment, as discussed above, there has been pronounced interest in developing alternative treatments in recent years, specifically the biological transformation of these pollutants as a green technology (Domaradzka et al., 2015). The future inclusion of bioremediation technologies in traditional WWTP treatments is progressive as it will result in the detoxification of hazardous substances, it is less disruptive to the environment than harsh oxidative chemicals, and more cost-efficient. With perseverance, research into optimization could result in the complete eradication of target pollutants, rooting out release into the environment.

The wastewaters containing PhACs and their metabolites reaching WWTPs are commonly treated via purification systems. The potential of drug remediation via biological treatment utilizing microbes has been demonstrated (Kebede et al., 2018). Biological systems are often used in conjunction with advanced treatments and combined with conventional activated sludge (CAS) systems due to limitations associated with the process (Crini and Lichtfouse, 2019). Advanced biological treatments include modified CAS, aerobic granular systems, moving bed bioreactors (MBBRs), anammox systems, and membrane bioreactors (MBRs) (Grassi et al., 2012). However, some of these processes, such as MBRs, could result in the generation of biosolids or sewage sludge as byproducts of required maintenance. Sewage sludge, after different stabilization processes such as thermophilic anaerobic digestion, continues onto different processes, such as composting, which could facilitate the transfer of PhACs and their metabolites into various trophic levels of the food web when used as a soil amendment (Marcoux et al., 2013).

Bioremediation, utilizing native microbial monocultures or consortia or bioaugmentation, has been used for decades as a sustainable technology to manage anthropogenic pollution (Ahumada-Rudolph et al., 2021). The advantages of bioremediation include less input of hazardous chemicals, energy, and time, and it is cheap relative to other technologies (Azubuike et al., 2016). The major benefit of bioremediation is that the pollutant is chemically transformed and not only shifted from one environment to another (Mashi, 2013). However, a significant criticism of bioremediation has been that the remediation speed does not meet the requirements for the treatment capacity. Nonetheless, considering the benefits of the approach, attempts on optimizing the efficiency and decreasing retention times are being made and are reviewed below for mycoremediation. Developments in phyto- and phycoremediation of pharmaceuticals have been reported and recently reviewed (Vilvert et al., 2017; Rao et al., 2019; Kaloudas et al., 2021; Kurade et al., 2021) and thus, not included here.

Bacterial remediation has been reviewed to some extent (Shah and Shah, 2020), and, therefore, a brief overview of previously undiscussed advances are included here alongside mycoremediation. Bacterial communities have the ability to degrade and mineralize many xenobiotic compounds and have thus been used for centuries in wastewater-activated sludge (Xu et al., 2018). Bioremediation technologies have been advanced by studies elucidating the importance of facilitating biofilm growth in achieving maximum efficiency and community stability and survival (Edwards and Kjellerup, 2013). The majority of the available literature on bacterial remediation has focused on the aerobic degradation of pharmaceuticals by individual bacteria or consortia in which oxygenases are reported to be involved (Ferreira et al., 2018). Activated sludge, in which an uncharacterized bacterial consortium in suspension is responsible for the remediation, is one of the most widely used biological methods to treat pharmaceutical wastewater at a large scale (Bis et al., 2019). However, due to operational issues associated with the development of large amounts of sludge, research has been invested in developing bespoke bacterial consortia for remediation, including microalgae and bacterial-microalgae consortia (Mamta et al., 2020).

In the environment, fungi are excellent decomposers through the nonspecific nature of enzymes, both intracellular and extracellularly secreted, which exhibit significant capabilities to degrade organic material (Rouches et al., 2016). More specifically, the ligninolytic (including peroxidases and laccases) and cytochrome P450 systems have been proven to be involved in the exceptional capacity of white-rot fungi to degrade recalcitrant pollutants (Park and Choi, 2020). The nonspecific nature of these enzymes also makes them an ideal approach to deal with the diverse chemical structures of the many classes of pharmaceuticals. Many fungal species are also hyperaccumulators, capable of absorbing and bioaccumulating xenobiotics from their environment, as demonstrated by the ability of mushrooms (Braeuer et al., 2020). Furthermore, fungi are known for their capacities to adapt to severe environmental constraints (Jiao and Lu, 2020), making them more tolerant to environmental changes than other bioremediation organisms. Thus, mycoremediation, which results in the reduced toxicity of wastewater (Jelic et al., 2012; Akhtar and Mannan, 2020), offers a comparatively cost-effective, eco-friendly, and effective approach to pollution remediation.

Macromycetes, aka mushrooms or polypores, were previously proven efficient in remediating various pharmaceuticals (Migliore et al., 2012; Cruz-Morató et al., 2014), including β-blockers and psychoactive drugs, anti-inflammatory drugs, antibiotics and hormones (Table 3). Mostly, investigations into the efficiency of fungi to remediate pharmaceuticals have been performed in flask batch experiments with white-rot fungi, especially *Trametes versicolor*, which exhibited impressive capacities for eliminating a vast range of pharmaceuticals. In bioreactors-based studies, *T. versicolor* was equally efficient, able to degrade various pharmaceuticals, including codeine, diazepam, carbamazepine, and metoprolol (Asif et al., 2017). The role of redox-mediators has also been extensively studied in improving the performance of laccase-based treatments (Ashe et al., 2016; Shao et al., 2019), including the treatment of pharmaceuticals (Nguyen et al., 2013; Vasiliadou et al., 2019). Studies employing filamentous micromycetes have shown potential for pharmaceutical remediation from wastewaters as reviewed by Olicón-Hernández et al. (2017) but are limited compared to the literature on macromycetes (Table 3). The efficiency of bacteria and fungi to remediate different classes of pharmaceuticals is discussed in more detail below.

**TABLE 3:** Summary of fungal remediation studies on the removal efficiency of single PhAC.

Pharmaceutical	Species	Experimental type	Contact time (days)	Start conc (mg/L)	Efficiency (%)	References
***Macromycetes***						
**Carbamazepine**	*Trametes versicolo*r	Lab, flask	6	9	94	Jelic et al., 2012
			7	0.05	61	
	*T. versicolo*r	Air pulsed fluidized bed reactor-batch	2	0.2	96	Jelic et al., 2012
	*T. versicolo*r	Air pulsed fluidized bed reactor–cont.	25	0.2	54	
	*Pleurotus ostreatus*	Lab, flask	7	0.04	68	Buchicchio et al., 2016
**Diclofenac**	*T. versicolor*	Cont. membrane reactor	1	0.3-1.5	55	Yang et al., 2013
**Ofloxacin**	*T. versicolor*	Lab, flask	7	10	80	Gros et al., 2014
		Fluidized air pulse bioreactor sterile	8	0.03	98.5	
		Fluidized air pulse bioreactor nonsterile	5	0.003	99	
	*Irpex lacteus*	Lab, flask	10	10	100	Čvanv̌arová et al., 2015
**Cefuroxime axetil**	*Imleria badia*	Lab, flask	7	400, 1000, 1600	100	Dąbrowska et al., 2018
	*Lentinula edodes*	Lab, flask	7	400, 1000, 1600	100	
**Oxacillin**	*Leptosphaerulina* sp.	Lab, flask	6	16	100	Copete-Pertuz et al., 2018
**Cloxacillin**	*Leptosphaerulina* sp.	Lab, flask	7	17.5	100	
**Dicloxacillin**	*Leptosphaerulina* sp.	Lab, flask	8	19	100	
**Clarithromycin**	*P. ostreatus*	Lab, flask	7	0.00003	55	Buchicchio et al., 2016
**Oxytetracycline**	*P. ostreatus*	Lab, flask	14	50, 100	100	Migliore et al., 2012
**Flumequine**	*I. lacteus*	Lab, flask	10	10	100	Čvanv̌arová et al., 2015
**Ciprofloxacin**	*I. lacteus*	Lab, flask	10	10	100	
**Testosterone**	*L. edodes*	Lab, flask	21	100000, 200000	100	Muszyńska et al., 2018
**17α-Ethinylestradiol**	*L. edodes*	Lab, flask	21	400, 800	100	
	*L edodes (stalk)*	Bioabsorption	0.02	2	100	de Jesus Menk et al., 2019
	*L. edodes (substrate)*	Bioabsorption	0.02	2	80	
	*Agaricus bisporus (stalk)*	Bioabsorption	0.02	2	100	
***Micromycetes***						
**Carbamazepine**	*Trichoderma harzianum*	Lab, flask	7	0.004	72	Buchicchio et al., 2016
	*Phanerochaete chrysosporium*	Bioreactor, nonsterile	100	5	80	Zhang and Geißen, 2012
		Continuously stirred bioreactor	50	0.5	63	Rodarte-Morales et al., 2012b
**Diclofenac**	*Penicillium oxalicum*	Lab, flask	1	29	100	Olicón-Hernández et al., 2019
	*Mucor hiemalis*	Lab, flask	6	0.05	97	Esterhuizen-Londt et al., 2017
	*P. chrysosporium*	Fed-batch stirred bioreactor	1	0.8	99	Rodarte-Morales et al., 2012a
		Continuously stirred bioractor	1	1	93	Rodarte-Morales et al., 2012b
**Acetaminophen**	*M. hiemalis*	Lab, flask	1	0.02	< 50	Esterhuizen-Londt et al., 2016b,a
	*P. chrysosporium*	Lab, flask	7	0.25	99	Esterhuizen et al., 2021
**Ibuprofen**	*P. chrysosporium*	Fed-batch stirred bioreactor	0.63	0.8	99	Rodarte-Morales et al., 2012a
		Continuously stirred bioractor	1	1	93	Rodarte-Morales et al., 2012b
**Naproxen**	*P. chrysosporium*	Fed-batch stirred bioreactor	1	0.8	99	Rodarte-Morales et al., 2012a
		Continuously stirred bioractor	3	1	90	Rodarte-Morales et al., 2012b
**Clarithromycin**	*T. harzianum*	Lab, flask	7	0.00003	57	Buchicchio et al., 2016
**Oxytetracycline**	*Penicillium commune*	Lab, flask	15	250	68	Ahumada-Rudolph et al., 2021
	*Epicoccum nigrum*,	Lab, flask	15	250	76	
	*Trichoderma harzianum*	Lab, flask	15	250	77	
	*Aspergillus terreus*	Lab, flask	15	250	74	
	*Beauveria bassiana*	Lab, flask	15	250	78	
**Erythromycin**	*Penicillium oxalicum* RJJ-2	Lab, flask	4	100	84	Ren et al., 2021
**17 β-estradiol (E2)**	*Trichoderma citrinoviride AJAC3*	Lab, flask	4	200	100	Chatterjee and Abraham, 2019

### Beta-Blockers and Psychoactive Drugs

Carbamazepine, which is not adequately eliminated via standard wastewater treatments and is thus frequently detected in the environment (Ekpeghere et al., 2018), has been reported to be degraded by the macromycete *T. versicolor*. By employing *T. versicolor*, Jelic et al. (2012) achieved 94% degradation of carbamazepine (9 mg/L) after six days in flask experiments. With a reduced concentration (50 μg/L), Jelic et al. (2012) reported a lower remediation percentage of 61% achieved in seven days. The same group evaluated the fungus’s remediation efficiency of carbamazepine in an air pulsed fluidized bed bioreactor operated in batch and continuous mode. In batch mode, 96% of the drug was eliminated after 2 days, with higher efficiency achieved in the bioreactor than in flasks explained by glucose addition, pH management and air supplementation. In continuous mode, carbamazepine was reduced by 54% in the outflow compared to the inflow concentration of 200 μg/L (Jelic et al., 2012). With *Pleurotus ostreatus*, another white-rot fungus, 68% carbamazepine was degraded in liquid culture after seven days with no further degradation after this time (Buchicchio et al., 2016).

The filamentous fungus *Trichoderma harzianum* was able to degrade 72% of environmentally detected concentrations of carbamazepine (4 μg/L) (Buchicchio et al., 2016), which was superior compared to the polypore *P. ostreatus*. In a non-sterile bioreactor, *Phanerochaete chrysosporium* was able to degrade up to 80% of 5 mg/L carbamazepine when supplied with a diluted synthetic feed (Zhang and Geißen, 2012). In a fed-batch stirred bioreactor, *P. chrysosporium* removed yo to 60% carbamazepine (0.5 mg/L); however, it was unable to degrade diazepam (0.25–0.5 mg/L) (Rodarte-Morales et al., 2012a). In a fixed bed reactor, where the pellets of *P. chrysoporium* were immobilized in polyurethane, the remediation efficiency of carbamazepine and diazepam was significantly improved (Rodarte-Morales et al., 2012b).

Even though nearly complete remediation of some beta-blockers and psychoactive drugs could be achieved in flask and lab bioreactor scale experiments, large or even pilot scale studies are needed to comprehensively evaluate the effect of upscaling on the remediation efficiency and the cost-effectiveness of using fungi for these drugs as an add-on treatment in WWTPs.

### Non-steroidal Anti-inflammatory Drugs and Analgesics

Bioremediation using bacterial monocultures for the treatment of NSAIDs has not to date been successful (Wojcieszyńska et al., 2014). Some studies have shown the elimination of NSAIDs by bacterial consortia in WWTPs. One study showed that eliminating acetaminophen in an MBR was mainly associated with heterotrophic bacteria. They concluded that using a microbial consortium in an MBR could be complimentary for post-treating effluents from treatment plants containing pharmaceutical products (De Gusseme et al., 2011). However, as seen with the consortia in CAS treatments, which are unidentified and often change in conjunction with the wastewater being treated, consortia in bioreactors may also change, resulting in decreased efficiency. To further explore the use of bacterial consortia in bioreactors, long-term studies need to be conducted on-site in WWTPs to evaluate the composition and stability of the bacterial assemblage, and it should be modeled how shifts could influence remediation.

In terms of mycoremediation, *T. versicolor* has shown very promising results in the remediation of NSAIDs (Asif et al., 2017; Tińma et al., 2021). In a continuous MBR (with a hydraulic retention time of one day), *T. versicolor* eliminated 55% of diclofenac added at concentrations ranging from 0.3 to 1.5 mg/L (Yang et al., 2013). Another fungus that demonstrated the potential to degrade anti-inflammatory drugs is the edible fungus *Lentinula edodes* (shiitake mushroom). The degradation products of piroxicam produced by *L. edodes* degradation has already been described (Muszyńska et al., 2019); however, the remediation percentage was not reported.

*Penicillium oxalicum* was capable of totally degrading diclofenac in 24 h, starting from an initial concentration of 29.6 mg/L (100 μM) (Olicón-Hernández et al., 2019). For *Mucor hiemalis* f. *irnsingii* (DSM 14200; Zygomycota), a strain isolated from a groundwater source in Germany, the diclofenac (10–50 μg/L) removal percentages ranged between 90 and 97% after 6 days (Esterhuizen-Londt et al., 2017). The same micromycete was also employed for the remediation of acetaminophen. After 24 h of exposure to environmentally relevant concentrations of acetaminophen (up to 20 μg/L), *M. hiemalis* was able to degrade up to 50% (Esterhuizen-Londt et al., 2016b,a). However, after 24 h, diclofenac remediation halted; nevertheless, pH maintenance could overcome this (Esterhuizen et al., 2021). The acetaminophen remediation efficiency of *Phanerochaete chrysosporium* (97 and 99% of 250 μg/L APAP after 3 and 7 days, respectively) was far superior to that of *M. hiemalis*, and co-cultivation of the two species resulted in a decreased remediation efficiency compared to *P. chrysosporium* in single (Esterhuizen et al., 2021).

Furthermore, Olicón-Hernández et al. (2020) studied the degradation of a mixture of acetaminophen, diclofenac, ibuprofen, ketoprofen and naproxen with *P. oxalicum*, starting from an initial concentration of 50 μM of each compound in both flasks and bench fluidized bioreactors. *P. oxalicum* showed higher degradation percentages in the bioreactor than at the flask scale. The authors reported that with glucose addition in the fluidized bed bioreactor, degradation of all drugs was complete after eight days (Olicón-Hernández et al., 2020).

In a fed-batch stirred bioreactor, *P. chrysosporium* oxidatively degraded up to 99% of diclofenac, ibuprofen, and naproxen each at a concentration of 0.8 mg/L (Rodarte-Morales et al., 2012a). However, in continuously stirred bioreactors, *P. chrysosporium* degraded diclofenac, ibuprofen, and naproxen (1 mg/L each) up to 95%.

With these preliminary flask and laboratory-scale reactor experiments, the potential of using mycoremediation to treat NSAIDs is highlighted. However, data on the performance of the fungi in WWTPs is lacking, making a consequential evaluation impossible. A potential issue that may arise in practice is the need for maintenance and controlled conditions, as highlighted by the study conducted by Esterhuizen et al. (2021), which showed the need for maintaining pH conditions.

To overcome the limitations of monocultures for the remediation of these pollutants, the use of microorganism-consortia has been explored. Consortia of microorganisms that complement each other could improve biological wastewater treatment technologies significantly. For example, Nguyen et al. (2013) found that a mixed bacterial culture in conjunction with *T. versicolor* in an augmented MBR better degraded PhACs than a system containing the fungus or bacteria alone (Nguyen et al., 2013). In addition, bioaugmentation technologies using adapted fungi, such as *P. oxalicum*, have proven an interesting technology to overcome the problem of competition with autochthonous microbiota, as demonstrated by Olicón-Hernández et al. (2021). However, more data are needed to define complementary species since the study by Esterhuizen et al. (2021) revealed that co-culture of certain species could reduce the remediation efficiency.

### Antibiotics

In general, low remediation efficiencies for most antibiotics from wastewaters have been reported using CAS treatment (Chaturvedi et al., 2021a; Zou et al., 2022). Thus, CAS could be applied to treat some antibiotics; however, not all. More recently, increased antibiotic removal percentages have been reported with anoxic/anaerobic/oxic granular and suspended activated sludge processes, specifically with sulfamethoxazole (Kang et al., 2018). The shortcoming could be improved by supplementing the sludge with bacteria capable of better remediation or even mixing treatments and complementing CAS with mycoremediation with macromycetes has been proven to be very effective for antibiotics.

*T. versicolor*, in flask experiments, degraded the antibiotic ofloxacin (10 mg/L) with 80% efficiency. When upscaled to 10 L fluidized air-pulse bioreactors, ofloxacin spiked into hospital waste was removed by 98.5% under sterile conditions and 99% under nonsterile conditions (Gros et al., 2014).

Buchicchio et al. (2016) reported the elimination of 55% clarithromycin (0.03 μg/L) by edible mushroom *P. ostreatus* and 57% by the micromycete *T. harzianum*. In flask experiments, *P. ostreatus* could also eliminate oxytetracycline (50 and 100 mg/L) after 14 days (Migliore et al., 2012). The antifungal drugs bifonazole and clotrimazole were also bioaccumulated and eliminated by the mycelia of the edible fungus *Lentinus edodes* (Kryczyk-Poprawa et al., 2019). In flask experiments, the cephalosporin antibiotic cefuroxime axetil was entirely eradicated by both the edible mushrooms *Imleria badia* and *L. edodes* within seven days at all concentrations tested (400, 1,000, 1,600 mg/L) (Dąbrowska et al., 2018).

*Leptosphaerulina* sp. removed oxacillin (16 mg/L, in 6 days), cloxacillin (17.5 mg/L, in 7 days) and dicloxacillin (19 mg/L, in 8 days) from water in flask experiments by the action of laccase and peroxidase. With synthetic hospital waste, oxacillin was reduced by 60% within two days and wholly eradicated after six days by the *Leptosphaerulina* sp. (Copete-Pertuz et al., 2018).

In a comparative study investigating the degradation efficiencies of five ligninolytic fungi, the polypore *Irpex lacteus* degraded the fluoroquinolone antibiotic flumequine, ciprofloxacin and ofloxacin effectively within six days (Èvanèarová et al., 2013; Čvanv̌arová et al., 2015). *I. lacteus* also removed the residual antibacterial activity of norfloxacin and ofloxacin via the action of manganese peroxidase (Čvanv̌arová et al., 2015).

Ahumada-Rudolph et al. (2021) evaluated fifty fungal isolates from sediments of salmon hatcheries for their oxytetracycline remediation abilities. The filamentous fungi *Penicillium commune*, *Epicoccum nigrum*, *T. harzianum*, *Aspergillus terreus*, and *Beauveria bassiana* were identified as having the best remediation rates amounting to a maximum of 78% removal of a 250 mg/L oxytetracycline concentration in flask experiments (Ahumada-Rudolph et al., 2021). *P. oxalicum* RJJ-2 has also been studied in the degradation of erythromycin and degraded 84.88% erythromycin after 96-h incubation used as the sole carbon source producing different metabolites (Ren et al., 2021).

The studies on the efficiency to remove antibiotics reported to date have focused on the efficiency under set conditions. However, in a WWTP, environmental conditions and even the water’s parameter would fluctuate from time to time. How this could affect the remediation efficiency and fungal longevity over time is unknown. Nevertheless, this information could be essential in evaluating this technique’s applicability in the field. It is importante to note the relevance of the use of fungi in removing antibiotics since bacteria can adquire rapidly antibiotic resistance genes during bioremediation and contribute to the widespread of ARGs.

### Endocrine Disruptors

The fate of estrogenic hormones treated via activated sludge systems in full-scale WWTPs was reviewed by Hamid and Eskicioglu (2012). Activated sludge systems with nutrient removal achieved more than 90% degradation in most studies (Hamid and Eskicioglu, 2012).

Degradation of testosterone and 17α-ethinylestradiol (EE2) by the fungus *L. edodes* was reported by Muszyńska et al. (2018), with no testosterone or 17α-ethynylestradiol detected after 21 days (Muszyńska et al., 2018). Interestingly, the white-rot fungus *P. ostreatus* HK 35, in the presence of the natural water microbiota of a WWTP, degraded up to 90% of 17β-estradiol (E2) within 12 days in various bioreactor sizes and under different regimes (Křesinová et al., 2018). The micromycete *Trichoderma citrinoviride* AJAC3 degraded 99.6% 17 β-estradiol (E2) (at a starting concentration of 200 mg/L) after four days attributed to the secretion of ligninolytic enzymes (Chatterjee and Abraham, 2019). A study investigating the efficiency of mycoremediation to remove 17 β-estradiol (E2) from poultry litter found that the polypore *Pycnoporus* sp. SYBC-L3 could remove up to 78.4% via solid-state cultivation supplemented with citric acid and lignocellulosic biomasses to boost laccase activity (Liu et al., 2016), an approach that could be tested for increasing remediation from wastewaters.

Even though the hormone remediation percentage reported with mycoremediation is, in some cases, higher than the CAS studies reviewed by Hamid and Eskicioglu (2012), a comparison is not possible since the studies on the fungal efficiency were performed in the laboratory in comparison to the CAS studies completed on-site at WWTPs. In addition to excluding several variables that could impact the remediation efficiency, these studies have established the remediation efficiencies for individual compounds. In wastewater effluent, a mixture of not only PhACs are present, and the synergistic effect of all these compounds could affect the efficiencies reported (Chatterjee and Abraham, 2019).

Bioabsorption is another approach to PhAC remediation with fungi. *L. edodes* and *Agaricus bisporus* (champignon) stalks removal 100% of 17α-ethinylestradiol (EE2) in 20 and 30 min, respectively via absorption, whereas Shiitake substrate absorbed 80% (de Jesus Menk et al., 2019).

Despite the high hormone remediation percentages achieved with fungi described above, few studies have been published on this topic in the last decade, and renewed investigations would greatly benefit the development of this technique to elevate the environmental impacts of hormones released untreated from WWTPs.

### Mixed Effluents

Cruz-Morató et al. (2013) studied the degradation of pharmaceuticals in hospital effluent by *T. versicolor*. By employing fluidized bed bioreactor in fed-batch mode, *T. versicolor* could eliminate ibuprofen (2.34 mg/L), acetaminophen (1.56 mg/L), ketoprofen (0.08 mg/L), propranolol (0.06 mg/L), and azithromycin (4.31 mg/L). By running the fluidized bed reactor in continuous mode, the efficiency was increased, and the fungus was able to completely remove acetaminophen (109 mg/L), naproxen (1.62 mg/L), ibuprofen (35.5 mg/L), diclofenac (0.477 mg/L), codeine (0.606 mg/L), trimethoprim (0.853 mg/L), and sulfamethoxazole 1.41 mg/L 100%, and partially remove several other drugs. However, salicylic acid, tetracycline, and carbamazepine were not degraded (Cruz-Morató et al., 2013, 2014). *T. versicolor* was also investigated for its performance to remediate PhACs from veterinary hospital wastewater; however, only 66% removal efficiency was achieved in a non-sterile batch bioreactor (Badia-Fabregat et al., 2016).

*P. oxalicum* XD.3.1 has also been used in batch bench-scale bioreactors to test the remediation efficiency with real hospital effluents. Within 24 h, *P. oxalicum* was able to reduce the majority of the PhAC present in the effluent, including ketoprofen, naproxen and paracetamol. Interestingly, *P. oxalicum* also affected the native microbiota, including opportunistic pathogens (Olicón-Hernández et al., 2021). In fluidized bed bioreactor studies, including hospital wastewater spiked with 10 mg/L each diclofenac, ketoprofen, and atenolol, *P. ostreatus* completely remediated diclofenac in 24 h and 50% of the ketoprofen in 5 days. However, atenolol was not removed (Palli et al., 2017). These studies demonstrated the complexity of degrading PhAC in mixed matrix effluents, which could drastically reduce the remediation efficiency. Therefore, more studies should be conducted at a larger scale employing real effluents to develop mycoremediation using fungi.

Currently, mycoremediation studies on other emerging PhACs, such as anticancer and antiretrovirals, are lacking. Testing fungal species capable of degrading pharmaceuticals at a laboratory scale is ongoing; however, it is difficult to predict how biological organisms would cope in a treatment facility exposed to chemical mixtures over long periods. Thus, recognizing the potential of mycoremediation for the treatment of pharmaceuticals demonstrated to date, studies regarding functioning and long-term applicability in practical terms to evaluate the feasibility of mycoremediation fully are still lacking. However, limitations such as partial degradation of pharmaceuticals and reduced efficiency at lower PhAC concentrations have been identified but could be overcome by using consortia or optimizing enzyme extraction and isolation to reduce costs.

The exact mechanism of degradation for each fungal type and PhACs is still vague due to its complexity and all the counterparts involved (Dąbrowska et al., 2018). However, the degradation seems to include activities of the intracellular enzymatic system such as the cytochrome P450 system, mainly in fungi lacking ligninolityc enzymes, and the extracellular enzymatic system, including lignin peroxidase, manganese peroxidase, laccase, versatile peroxidase as well as hydroxyl and free radical, in the case of lignin degrading enzymes producers (Dąbrowska et al., 2018; Barh et al., 2019). Nevertheless, elimination is reported to produce no toxic byproducts (Copete-Pertuz et al., 2018), therefore necessitating further studies into mycoremediation optimization for an add-on in WWTPs and elucidating the mechanism of action.

## Isolated Fungal Enzymes

The use of isolated fungal enzymes could also overcome some limitations associated with mycoremediation. Fungal enzymes, specifically the ligninolytic enzymes, have been recognized for their abilities to transform a broad range of recalcitrant PhACs. However, difficulties in growing fungi on a large scale, together with the long incubation processes, extensive growth phase, and spore formation, have prompted the exploration of extracted crude and isolated enzymes (Stadlmair et al., 2018). Though, to date, the main limiting factor has been the high cost of the enzyme purification procedure.

Commercially available laccases from *T. versicolor* efficiently degraded diclofenac, trimethoprim, carbamazepine and sulfamethoxazole as individual drugs, but the remediation efficiency decreased when applied to mixtures of the drugs (Alharbi et al., 2019). Kang et al. (2021) isolated laccases from *Bjerkandera* spp., which could efficiently remediate acetaminophen under a range of pH conditions (Kang et al., 2021). In a study employing immobilized laccases from *Trametes hirsuta*, Hachi et al. (2017) reported better remediation efficiencies for carbamazepine and acetaminophen (40 and 70%) in single compared to in mixtures (5 and 25%) (Hachi et al., 2017).

Using laccases (2,000 U/L) isolated from *Myceliophthora thermophile*, 94.1 and 95.5% of estrone E1 and 17β-estradiol E2 could be degraded within 8 h in the presence of a natural mediator in a fed-batch bioreactor. In an enzymatic membrane reactor (EMR) with a stir-tank configuration, this percentage was increased to 95% for E1 and near total E2 degradation (Lloret et al., 2010). This indicates that the bioreactor type significantly impacts the remediation efficiency regarding isolated enzymes. In a study by Becker et al. (2017), immobilized laccase from *T. versicolor* and *M. thermophila* could degrade 83 and 87%, respectively, of estrogenic compounds (E1 estrone; E2 17β-estradiol; EE2 17α-ethinylestradiol) in mixtures with other endocrine-disrupting compounds within 6h (Becker et al., 2017). Golveia et al. (2018) reported 96.5% remediation of 10 mg/L 17-α-ethinylestradiol by *Pycnoporus sanguineus* laccase (1,642 U/mL) after 8 h (Golveia et al., 2018). It would be noted that 1% (v/w) was added to the fungal culture to promote optimal laccase production concentration before extraction.

Utilizing isolated enzymes has the advantages of reducing the remediation time by avoiding the lag phase of fungal growth, reducing sludge production, and facilitating process control (Jebapriya and Gnanadoss, 2013). Apart from the high cost as a disadvantage, a study by Nguyen et al. (2014) demonstrated another drawback of using isolated enzymes (Nguyen et al., 2014). In a direct comparison, whole-cell culture degraded trace organic compounds with higher efficiency, which is said to be facilitated by biosorption and the activity of both intracellular and mycelium associated enzymes.

## Conclusion

The environmental impact of pharmaceuticals and their proper elimination from wastewaters have gained interest in recent years, mostly due to the intrinsic characteristics of these compounds, their massive use, and the negative effects on the environment and humans. Although they are medicinal substances developed to aid in the well-being of organisms, their indiscriminate use can lead to irreversible environmental problems. Therefore, it is important to create legislation according to the current standards of using substances and eco-friendly trends. More versatile and efficient systems for eliminating PhACs such as mycoremediation are being developed to lessen or avoid the problems associated with pharmaceutical pollution in the environment. However, these promising techniques are still at a laboratory scale and data regarding the application in WWTPs are still lacking. Even though new techniques for the remediation of PhAC are being developed and optimized, relative to the development of new drugs, implementing these techniques into practice is slow. New promising approaches for this purpose, such as genetic engineering, are still in their infancy. Thus, the new editing tool, such as CRISPR-Cas9, could help to introduce metabolic genes focused on target recalcitrant compounds. Much more studies are still necessary to deal with the problem of PhACs.

## Author Contributions

ME, EA, DRO-H: conceptualization. MO and ME: literature search and data analysis and original draft preparation. MO, ME, DRO-H, JG-L, and EA: critical revision of the work. All authors contributed to the article and approved the submitted version.

## Conflict of Interest

The authors declare that the research was conducted in the absence of any commercial or financial relationships that could be construed as a potential conflict of interest.

## Publisher’s Note

All claims expressed in this article are solely those of the authors and do not necessarily represent those of their affiliated organizations, or those of the publisher, the editors and the reviewers. Any product that may be evaluated in this article, or claim that may be made by its manufacturer, is not guaranteed or endorsed by the publisher.
