# Supplementary material for: Pharmaceutical Pollution in Aquatic Environments: A Concise Review of Environmental Impacts and Bioremediation Systems
Source: Front Microbiol. 2022 Apr 26;13:869332. doi: 10.3389/fmicb.2022.869332 (PMC9087044; doi:10.3389/fmicb.2022.869332)
Supplement: Supplementary file 1 [file Data_Sheet_1.docx]

Supplementary Material


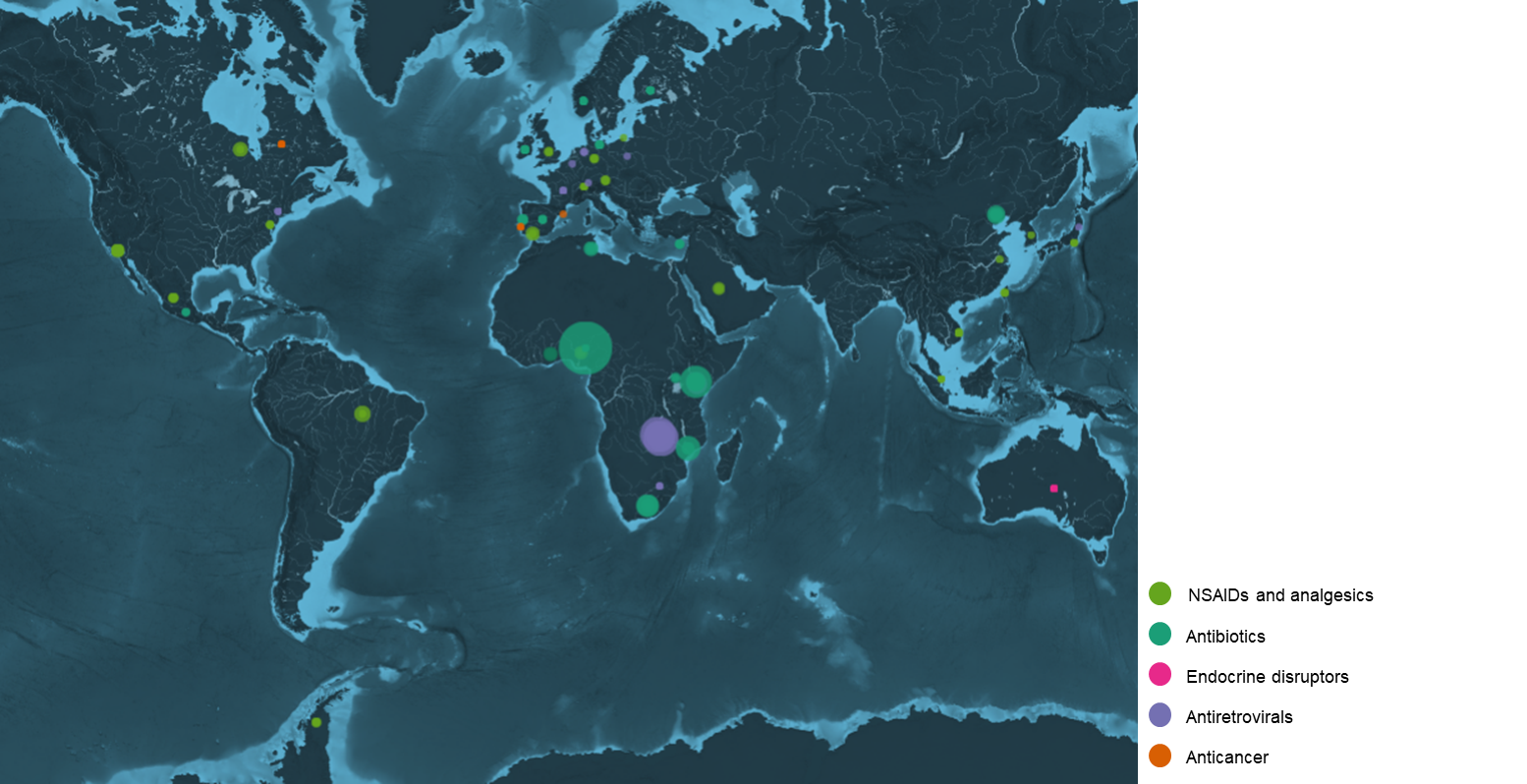


**Figure S1.** Global distribution of pharmaceuticals. Map showing the concentrations and distribution of different types of pharmaceuticals: NSAIDs and analgesics, antibiotics, endocrine disruptors, antiretrovirals and anticancer drugs. Larger dots indicate higher maximum concentrations recorded. To view the interactive map in detail, visit the following link (<https://public.flourish.studio/visualisation/6821658/>).
